# CD38+ Alveolar macrophages mediate early control of M. tuberculosis proliferation in the lung

**DOI:** 10.21203/rs.3.rs-3934768/v1

**Published:** 2024-07-15

**Authors:** David Russell, Davide Pisu, Joshua Mattila, Luana Johnston

**Affiliations:** Cornell University; Cornell University; University of Pittsburgh; Cornell University

## Abstract

Tuberculosis, caused by *M.tuberculosis* (Mtb), remains an enduring global health challenge, especially given the limited efficacy of current therapeutic interventions. Much of existing research has focused on immune failure as a driver of tuberculosis. However, the crucial role of host macrophage biology in controlling the disease remains underappreciated. While we have gained deeper insights into how alveolar macrophages (AMs) interact with Mtb, the precise AM subsets that mediate protection and potentially prevent tuberculosis progression have yet to be identified. In this study, we employed multi-modal scRNA-seq analyses to evaluate the functional roles of diverse macrophage subpopulations across different infection timepoints, allowing us to delineate the dynamic landscape of controller and permissive AM populations during the course of infection.

Our analyses at specific time-intervals post-Mtb challenge revealed macrophage populations transitioning between distinct anti- and pro-inflammatory states. Notably, early in Mtb infection, CD38^−^ AMs showed a muted response. As infection progressed, we observed a phenotypic shift in AMs, with CD38^+^ monocyte-derived AMs (moAMs) and a subset of tissue-resident AMs (TR-AMs) emerging as significant controllers of bacterial growth. Furthermore, scATAC-seq analysis of naïve lungs demonstrated that CD38^+^ TR-AMs possessed a distinct chromatin signature prior to infection, indicative of epigenetic priming and predisposition to a pro-inflammatory response. BCG intranasal immunization increased the numbers of CD38^+^ macrophages, substantially enhancing their capability to restrict Mtb growth.

Collectively, our findings emphasize the pivotal, dynamic roles of different macrophage subsets in TB infection and reveal rational pathways for the development of improved vaccines and immunotherapeutic strategies.

## Introduction

Tuberculosis (TB) remains a major global health issue, as reported in WHO’s Global Tuberculosis Report 2022^1^. *Mycobacterium tuberculosis* (Mtb), the etiological agent of TB, primarily infects the lungs and has evolved to survive the host immune response. Within the lungs, alveolar macrophages (AMs) play a critical role in protecting the airway surfaces upon early infection^2–5^, with recruited monocyte-derived macrophages increasing in number as the infection progresses^6,7^. Therefore, understanding the specific role of AMs during Mtb infection is key to uncovering disease mechanisms and guiding the development of effective vaccines.

In recent years, our understanding of the interactions between AMs and Mtb has grown considerably. Bulk transcriptomic studies, alongside dual RNA-seq, have demonstrated that AMs can facilitate Mtb replication and dissemination in the lung^6,8–10^. Recent advances in single-cell transcriptomics have uncovered the presence of heterogenous populations within the AM and IM lineages, each exhibiting varying responses to Mtb infection^9^. Moreover, innovative vaccination approaches, including subcutaneous, intravenous (iv) and pulmonary immunization using live BCG, have been found to activate AMs and innate immune cells, and provide lasting protection against Mtb^11–17^. However, despite these advancements, the specific AM populations and properties underlying this protective effect remain unidentified.

In this study, we leverage our established multi-modal single-cell RNA sequencing (scRNA-seq) protocol^9,18^ to identify the specific AM subsets that provide protection against Mtb. Employing the murine TB model, we evaluate macrophage phenotypes at 2-, 3-, 4-, and 6-weeks post-Mtb challenge to understand their roles in the early stages of infection and after BCG intranasal immunization.

Our findings traced the evolving lung immune response to Mtb infection. We observe macrophage populations transitioning from anti-inflammatory to pro-inflammatory states over time. Our analysis revealed the most pronounced phenotypic changes occurring within the resident AM populations and the recruited monocyte-derived AMs (moAMs), underscoring their critical roles in the immune response to Mtb infection. A crucial discovery was the identification of CD38, an extracellular NADase linked to macrophage activation, inflammation and infection^19–22^, as a potential marker for protective responses against Mtb. We observed distinct phenotypes for SiglecF^+^ CD38^+^ TR-AMs, SiglecF^−^ CD38^+^ moAMs, and SiglecF^+^ CD38^−^ TR-AMs throughout the disease process. Initially, CD38^−^ TR-AMs, displaying a muted pro-inflammatory response, were the main infected cell type. As infection progressed, CD38^−^ TR-AM numbers decreased, while CD38^+^ TR-AMs and the newly recruited moAMs rose in prevalence. In the later stages of infection, these CD38^+^ AM subsets emerged as the dominant infected host AM populations, demonstrating enhanced capacity to restrict Mtb’s growth.

Single-cell Assay for Transposase-Accessible Chromatin using sequencing (ScATAC-seq) analysis revealed that CD38^+^ TR-AMs have a unique chromatin structure that is distinct from CD38^−^ TR-AMs. These cells were already present in the lungs of naïve mice prior to infection, indicating an inherent epigenetic predisposition modulating their respective responses to Mtb infection. Additionally, intranasal immunization with live BCG, resulted in an increase in CD38^+^ macrophages in the lungs. This increase, the result of both an influx of CD38^+^ monocyte-derived macrophages and polarization of existing reactive pro-inflammatory CD38^+^ TR-AMs, offers significant protection against Mtb challenge.

To date vaccine development against tuberculosis has met with a modest success^23–26^ and it is seriously impaired by our lack of reliable biomarkers that are predictive of vaccine efficacy and outcome^27–31^. The identification and phenotypic characterization of those macrophage subsets best equipped to mediate protection against Mtb represents a significant advance in defining the immune response that we wish to drive with new immunotherapeutic interventions.

## Results and Discussion

### Ontogeny and intra-lineage diversity are key determinants in macrophage responses to Mtb infection.

**Figure 1:**
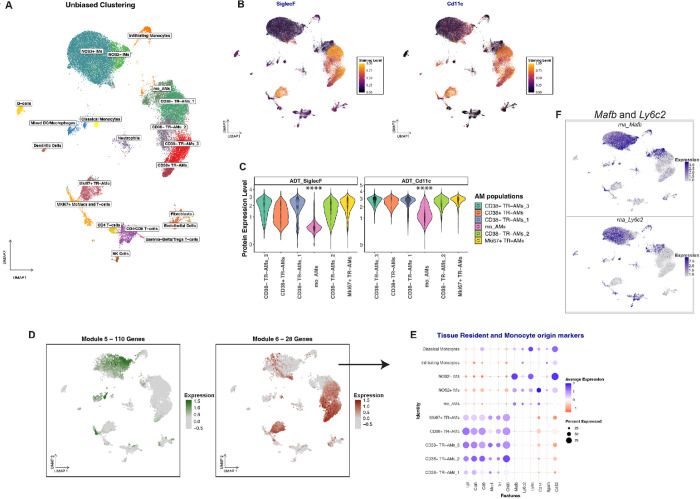
scRNA-seq Timepoint Analysis Reveals Distinct Macrophage Origins and Monocyte-to-Macrophage Differentiation Post-Mtb Challenge. **A:** UMAP visualization representing scRNA-seq data from 49,600 cells collected at 2-, 3-, 4-, and 6-weeks post-Mtb challenge. Cells are clustered based on their transcriptional profiles in an unbiased manner. **B:**UMAP visualizations of cells stained for SiglecF and Cd11c protein markers. **C:**Violin plot representations of protein expression levels for SiglecF and CD11c in different AM subsets. Statistical significance for each marker was tested using Kruskal-Wallis and post-hoc Dunn tests. Levels of significance are denoted as follows: *p < 0.05, **p < 0.01, ***p < 0.001, and ****p < 0.0001. **D:** Visualization of scWGCNA-derived gene modules expression levels mapped onto UMAP embeddings. **Left Panel:** Expression pattern of genes in Module 5 (110 genes). **Right Panel:**Expression pattern of genes in Module 6 (28 genes). **E:** DotPlot illustrating the expression pattern of tissue-resident and monocyte specific markers across macrophage cell clusters. Dot size and color intensity indicate percentage and average expression level, respectively. **F:** Umap plots showing expression levels (in log-normalized counts) for the *Mafb* (top) and *Ly6c2*genes (bottom) across all cells.

To better understand macrophage behavior during Mtb infection, we conducted an extensive multi-modal scRNA-seq analysis of macrophage phenotypes at 2-, 3-, 4-, 6-weeks post-Mtb challenge, which spans the transition from innate to adaptive immune responses^2,10,32–36^. Our aim was to determine how distinct macrophage populations respond post-Mtb infection, with the goal of identifying the subsets that are responsible for controller responses and those that favor Mtb replication and expansion. Consistent with our previous work^9^, we infected mice with the *hspx’*::gfp/smyc’::mCherry Mtb reporter strain. This strain expresses mCherry constitutively and GFP in response to host mediated immune-related stress^35^. For each infected host cell, we were able to assess the fitness status of intracellular Mtb, quantify the surface marker expression and profile their transcriptome^9^. After QC, our dataset comprised of 49600 cells, the significant majority of which were identified as macrophages (Figure 1A and Supp. Figure 1A).

Recent studies, including ours, have shown that both resident alveolar macrophages (AMs) and recruited interstitial macrophages (IMs), are ontogenically diverse ^6,8,37,38^ and comprised of phenotypically-distinct subpopulations^9,39–42^. These subpopulations exhibit markedly different inflammatory responses to Mtb infection^9^. In the current study, we extend these findings, further characterizing the discrete AM and IMs subpopulations based on their origin and inflammatory profiles.

**Figure 2:**
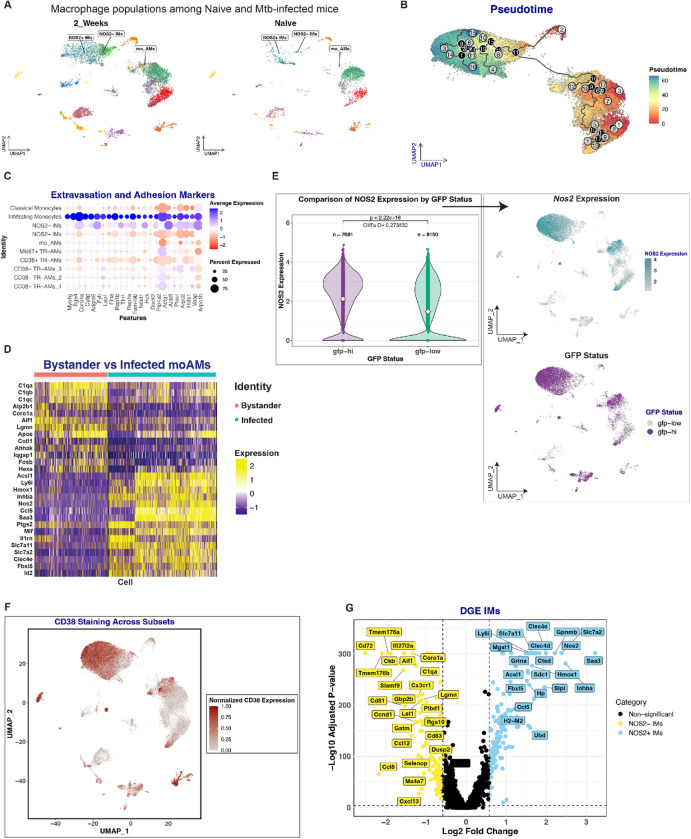
Identification of moAMs and temporal analysis of pro-inflammatory genes in AM and IM subsets. **A:** UMAP Visualization of cell clusters from Naïve and 2-weeks Post-infection timepoint. The NOS2^+^ IMs, NOS2^−^ IMs and moAMs clusters are highlighted. **B:** Pseudotime analysis of macrophages on the UMAP projection. Key features of the trajectory, including leaves (grey) and branch points (black), are annotated directly on the plot. **C:** DotPlot showing the expression levels of key extravasation and adhesion markers across various macrophage clusters. Dot size and color intensity indicate percentage and average expression level, respectively. **D:** Heatmap of differential gene expression for selected genes between bystander and infected moAMs. Each row represents a gene, and each column represents a cell. N = 1048 cells for moAMs Bystander and n = 1537 cells for moAMs Infected. **E: (Left Panel):** Violin plot showing the aggregate expression level of Nos2 based on GFP status. Statistical significance was assessed using a Wilcoxon test. Effect size measured as Cliff’s Delta is also displayed directly on the plot. **(Right Panel): UMAPs of infected cells**. In the top plot, cells are color-coded based on their Nos2 expression level, while in the bottom plot, they are color-coded based on their GFP status. **F:** UMAP of CD38 staining levels across cell subsets. **G:** Volcano plot showing differential gene expression in NOS2^+^ and NOS2^−^ IMs, with key genes of interest labeled.

AMs are often identified by their expression of surface markers SiglecF and CD11c^6,43,44^, and in our dataset we could identify AM subpopulations by SiglecF and CD11c staining (Figure 1B). Interestingly, we also noticed a population of cells that cluster with AMs by UMAP analysis, but do not stain for either SiglecF or CD11c (Figure 1A and 1C). To determine the origin of these cells, we focused on the early infection stages (Naïve and 2 weeks post-infection). We performed unbiased weighted gene correlation network analysis (WGCNA) on the resulting 13,125 cells to identify gene co-expression modules that define this population^45^. The analysis identified two gene expression modules that mirrored the differential staining of the SiglecF and CD11c antibodies (Figure 1D). Module 6 consists of a 28-gene co-expression set and includes genes commonly used to define tissue resident alveolar macrophages—such as *Cd9, Mrc1* (CD206*), Lpl, Trf, and Chil3* (Ym1) (Figure 1E)^8,9,46,47^. In contrast, Module 5, represented by a larger 110-gene set and expressed by the SiglecF/CD11c double negative population, is enriched in genes associated with the monocytic lineage (Figure 1E) and monocyte-to-macrophage differentiation (Supp Figure 1B). These AM-like cells express *MafB*—a driver of monocyte-to-macrophage differentiation—and *Ly6c2*, a marker for cells of monocyte origins (Figure 1F)^48–52^. We therefore designated this population as moAMs (monocyte-derived AMs). In the lungs of naïve mice moAMs are absent, implying these monocytes migrate there and differentiate to perform AM-related functions after Mtb infection (Figure 2A). Trajectory and pseudotime analyses corroborate this hypothesis, revealing that moAMs, along with the classical NOS2^+^ and NOS2^−^ IMs, represent distinct cell fates (grey leaves) that originate from a population of infiltrating monocytes (root 2 - white circle - and branching point 11 – black circle -), characterized by a unique transcriptional signature associated with leukocyte adhesion and extravasation (Figures 2B and 2C)^53–64^. Uninfected bystander moAMs express genes associated with macrophage functions including phagocytosis, lipid metabolism, and RNA/protein synthesis, indicative of roles beyond mere homeostasis (Figure 2D)^65–67^. This perspective is supported by the contrasting gene expression in Mtb-infected moAMs, which pivot to a pro-inflammatory state, as evidenced by the upregulation of genes such as *Acsl1, Hmox1, Saa3, Ptgs2, Slc7a11, Clec4e* among others(Figure 2D)^9^. This transition is marked by elevated *Nos2* expression and association with *hspx’*::GFP-high bacteria (Figure 2E). Such findings align with prior research which described the role of interferon-g in deactivating enhancer regions bound by the transcription factor Maf. This mechanism is crucial for suppressing M2 genes and increasing activation of monocyte-derived macrophages^68^. This moAM cohort expresses CD38 (Figure 2F), hence their designation as CD38^+^ moAMs.

Next, we focused on TR-AMs and identified two subpopulations based on their CD38 expression. The CD38^+^ population exhibits increased Nos2 expression and is associated with stressed (*hspx’*::GFP high) bacteria (Figure 2E and 2F). Notably, these TR-AMs are dual positive for SiglecF and CD11c (Figure 1B). They also express the TR-AM gene signature from Module 6 (Figure 1D), leading us to annotate them as CD38^+^ TR-AMs. Importantly, trajectory and pseudotime analyses establish the CD38^+^ TR-AMs as a distinct lineage from moAMs, as evidenced by their origin from root 1 (white circle) (Figure 2B). Additionally, we also identified CD38^−^ subpopulations of TR-AMs, associated with *hspx’*::GFP-low Mtb, indicating bacteria experiencing minimal host-derived stress (Figures 2E and 2F).

In examining the IM subpopulations, we observed a similar level of heterogeneity. While both IM populations appear to originate from infiltrating monocytes (Figure 2B), the NOS2^+^ IMs are CD38^+^, whereas the NOS2^−^ IM lack CD38 expression (Figure 2F). Looking at the transcriptional profiles of these populations revealed that CD38^+^ IMs, akin to CD38^+^ moAMs and TR-AMs, display a gene expression profile consistent with classic pro-inflammatory responses, which were previously linked to effective tuberculosis control^6,9^ (Figure 2G) (Suppl. Table 1).

Conversely, the IM subpopulation that is CD38^−^ not only lack Nos2 expression, but is also associated with *hspx’*::GFP-low Mtb (Figure 2E and 2F). Relative to NOS2^+^ IMs, these cells show amplified expression of genes linked to anti-inflammatory responses. This includes transcripts for complement proteins *C1q, Ccl8, Ms4a7* among others, which have been previously characterized (Figure 2G)^9,69–73^. Furthermore, this subset still shows a partial expression of extravasation and adhesion markers shared with infiltrating monocytes (Figure 2C). This suggests that they represent recently arrived interstitial monocytes that have just been infected and have not yet undergone immune activation (Suppl Table 1). In summary, our analysis identified distinct subpopulations of AMs and IMs with varying ontogeny and inflammatory profiles. Importantly, we discovered a population of monocyte-derived AMs capable of transitioning to a pro-inflammatory state upon Mtb infection, and two subpopulations of TR-AMs based on CD38 expression, which are associated with different bacterial phenotypes following Mtb infection.

### Pro-inflammatory immune responses are associated with rising CD38 expression in macrophages over time.

**Figure 3:**
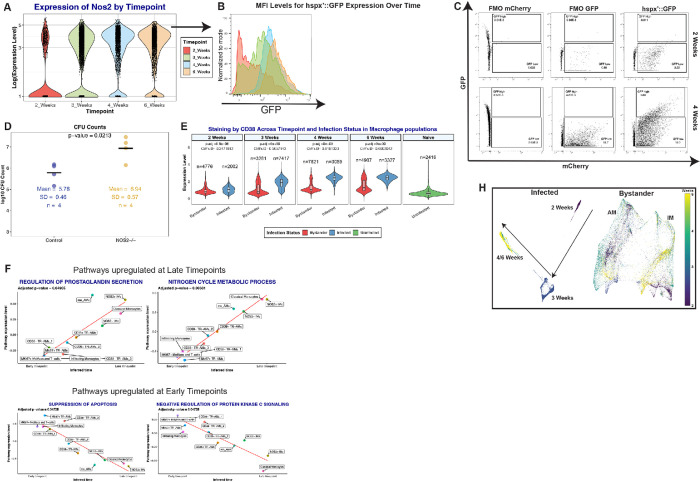
Pro-inflammatory immune responses are associated with rising CD38 expression over time. **A:** Violin plot displaying the distribution of *Nos2* gene expression levels in infected macrophages, split by timepoints. **B:** Overlaid flow cytometry histograms (on the left) and a bar chart (on the right) display the MFI of the Mtb GFP signal at various timepoints during the infection. **C:** Flow cytometry analysis of the Mtb smyc’::mCherry/hspx’::GFP reporter strain at 2 and 4 wpi in NOS2^−/−^ mice. **D:** Scatter plot comparing log10-transformed CFU counts between Control and NOS2^−/−^ mice. Each point represents an individual observation. Horizontal bars indicate the mean values. Summary statistics displayed for each group include the mean and standard deviation (SD), alongside the sample size (n). A t-test p-value annotation above the plot provides a statistical comparison of the means between the two groups. **E:** CD38 Protein Expression in macrophages, stratified by infection status and timepoint, displayed using violin and box plots with annotated adjusted p-values from pairwise t-tests for each timepoint. The effect size measured as Cliff’s Delta is also displayed directly on each plot, providing a standardized measure of the magnitude of differences. **F:** Pathways upregulated at late (top) and early (bottom) timepoints. Each cell cluster is represented by distinct colored points. Average pathway expression levels are on the y-axis, with inferred time from early to late on the x-axis. A red fitted line illustrates the expression trend over time. **G:** Scatter plot showing two-dimensional coordinates of single cells from a force-directed layout embedding for infected and bystander groups. Each point represents a cell, with its position on the plot determined based on its expression similarities to other cells. Colors indicate the time point of data collection as per the right-side color bar.

To understand the temporal evolution of macrophage immune responses following Mtb infection, we examined the expression patterns of pro-inflammatory gene signatures and associated markers over time. From our past studies, there was a discernible correlation between *Nos2* expression in host macrophages and bacterial stress (Figure 2E)^9^. Building on this, we tracked the temporal trends of *Nos2* expression within the infected macrophage populations. As the infection progresses, we observed an increase in nitric oxide production, reflected by both an increase in the level of *Nos2* expression per cell (intensity) and the number of cells expressing *Nos2* (prevalence), as shown in Figure 3A. Flow cytometry data confirmed this trend; the median fluorescence intensity (MFI) of the GFP signal from *hspx’*::GFP-infected cells increased over time, indicating heightened bacterial stress as the infection proceeded (Figure 3B). Importantly, infecting Nos2-KO mice with the *hspx’*::GFP Mtb reporter strain resulted in minimal GFP induction by Mtb at both 2 and 4 weeks post-infection (Figure 3C). The limited induction of GFP expression by Mtb in Nos2-KO mice was also accompanied by an increase in bacterial load (Figure 3D). Finally, flow cytometry analysis at 2 wpi of Rag1 and IFN-γ KO mice revealed no induction of *hspx’*::GFP (Supplementary Figure 2A), confirming that production of nitric oxide by pro-inflammatory macrophages is critically dependent on the adaptive immune response.

Consistent with these data, our surface marker analysis mirrored the trends observed in *Nos2* RNA levels. Specifically, CD38 protein levels – a marker associated with inflammation^20,22^ – increase in infected macrophages during the latter stages (Figure 3E). Parallel trends were also seen with the marker CD11b (Suppl Figure 2B). To gain deeper insights into the molecular mechanisms and biological processes driving these phenotypes, we performed a time-dependent pathway analysis^74^. This analysis identified 68 pathways that exhibited variations in expression as the infection progressed (p.adj < 0.05) (Table 1). Pathways showing increased representation over time were predominantly associated with inflammatory and anti-bacterial responses and were overly represented in CD38^+^ macrophages (Figure 3F and Supp figure 2C). Conversely, pathways that dominated early stages of infection were mostly associated with CD38^−^ macrophages, and aligned with processes linked to M2 polarization, tissue homeostasis and bacterial survival, such as negative regulation of protein kinase c signaling^75^, suppression of apoptosis and cell proliferation among others (Figure 3F and Supp figure 2D). Confirming these observations, our analysis revealed an increased recovery of Mtb mRNA reads from CD38^+^ macrophage populations over time, indicative of active bacterial degradation within these cells. Specifically, we noted low levels of Mtb reads at 2 weeks post-infection, which significantly increased from 3 to 6 weeks, as illustrated in Supplementary Figure 2E.

Intriguingly, when focusing on bystander macrophages, our analysis revealed no change in their CD38 staining levels, irrespective of the timepoint examined, contrasting starkly with their infected counterparts (Figure 3E). To validate this, we leveraged a force-directed layout to conduct unbiased graph-based clustering of both cell types^76^. While infected cells clustered according to duration of infection, bystander cells grouped based on their ontogeny without temporal distinctions (Figure 3G). This pronounced difference emphasizes that the inflammatory responses we observe aren’t merely a result of a broad immune activation of host macrophages over time; rather, they are finely tuned and specifically driven by active Mtb infection.

In conclusion, our findings highlight a dynamic shift in the phenotype and relative proportion of infected macrophage populations over the course of infection. This transition emphasizes the crucial role of CD38^+^ macrophages, which appear to be closely associated with the control of infection.

### The relative proportion of Mtb-infected cells shifts from AMs to IMs over time.

**Figure 4:**
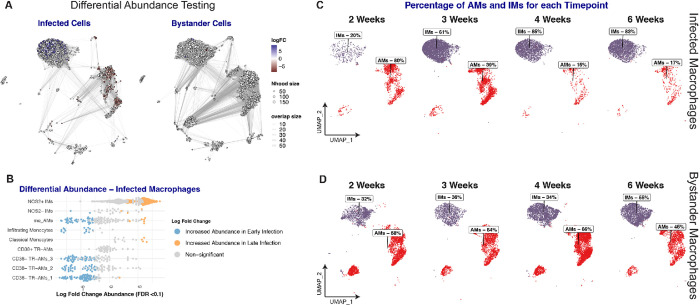
Integrative Analysis of Differential Abundance in MTB Infection. **A:** Neighborhood graph projected on the UMAP plot displaying cell cluster relationships by differential abundance in infected and bystander populations. Neighbors with significant Log2 Fold Change (LFC) depletion rates are in red, showing cells reduced during infection, while late-infection enriched neighbors are in blue (FDR < 0.1) **B:** Vertical scatter plot showing changes in macrophage subset abundance over time, with color-coded data points for statistical significance and change in direction: Blue for early infection increase (FDR < 0.1), Orange for late infection increase (FDR < 0.1), and Light Gray for no significant change. **C and D:** UMAP visualizations of macrophage subpopulations in infected (4C) and bystander (4D) cells across different timepoints of infection. The percentages of AMs and IMs within the total macrophage population at each timepoint are labeled on the plot.

To quantify the changes in the proportion of infected macrophage populations over time, we employed a recently developed statistical framework designed for differential abundance testing (DAB)^77^. We used this approach to assess whether changes in abundance of both infected and bystander cells occur over the course of infection. Our analysis identified variations in the abundance of 400 neighbors across different timepoints in infected AMs and IMs subpopulations (with FDR < 0.1). This contrasted with bystander populations that exhibited minimal variation (Figure 4A).

Mtb-infected neighbors belonging to the CD38^−^ TR-AMs subpopulations showed a marked decline in numbers late in infection. Conversely, neighbors within the NOS2^+^ IMs showed a significant increase in abundance during the same later timepoints (Figure 4B). Observations at the broader population level further highlighted this trend: at 2 weeks post-infection (wpi), AMs constituted 80% of all the infected macrophages, but by 3wpi this percentage was reduced to approximately 39%, and by the 4th and 6th wpi, it dropped to around 15% (Figure 4C). Overall, our data highlight a substantial shift in abundance from TR-AMs to IMs among the infected macrophages as the infection advances, as also confirmed by flow cytometry analysis (Supp. Figure 3). This trend sharply contrast the patterns seen in bystander AMs and IMs, whose proportions remain consistent across the analyzed timepoints (Figure 4A). The stability in the proportion of bystander macrophages in the Mtb-infected lung from the 2 weeks post-infection (wpi) suggests that, within our infection model, monocyte-derived cells have already been recruited to the lung by this time but remain largely uninfected (Figure 4D), similar to a previous report^78^.

This premise is further supported by examining the distinct timelines of infection rates between CD38^+^ IMs and moAMs. Despite pseudotime analysis indicating a shared origin for both populations from the early monocyte cluster (as illustrated in Figure 2B), CD38^+^ IMs only became the more abundant infected group by the 3rd week post-infection (Figures 4A and 4C). In contrast, a substantial proportion of CD38^+^ moAMs were infected at a much earlier stage, by 2wpi (Figures 4C and 2A). Recent studies provide context to these observations. The initial delay in the infection of monocyte-derived IM may be attributed to the early-stage preference of the infection for the lung’s alveolar space during the onset of tuberculosis. As the disease progresses, leading to the translocation of infected AMs from the alveolar to the interstitial lung space^2,6,8,10^, the infection rate of CD38^+^ *Nos2*^*+*^ IMs increases, leading to the overall shift in the abundance of infected macrophages from AMs to IMs as described above.

In summary, while the total bystander IM and AM populations in the lung remain constant from 2wpi onwards, our data indicates a decline in the number of newly infected TR-AMs as the infection shifts from the alveolar to the interstitial lung space. In the established infection, our data demonstrate that monocyte-derived IMs are the dominant host cell population accounting for the majority of new infection events.

### The early infection timepoints are dominated by CD38^−^ TR-AMs that are relatively nonresponsive to tuberculosis infection.

**Figure 5:**
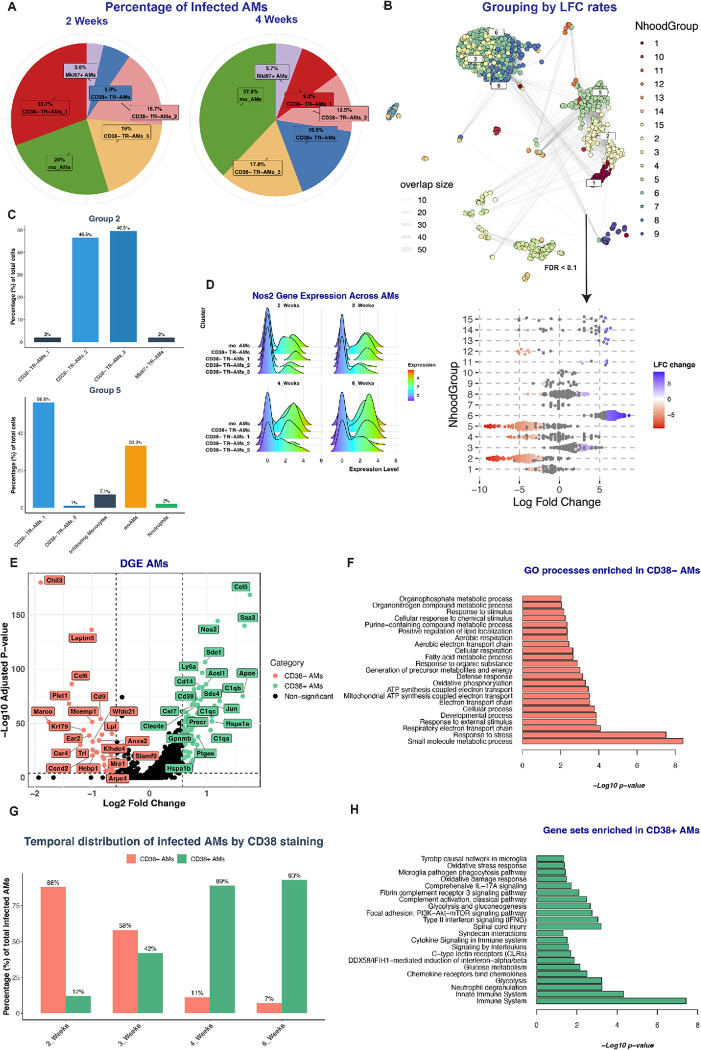
Analysis of CD38^+^ and CD38^−^ AMs post Mtb challenge. **A:** Pie charts of infected AM subtypes at 2 and 4 Weeks Post-Infection. **B:** (Top) UMAP visualization showing neighborhood (nhoods) clustering based on differential abundance, where each point represents a neighborhood group positioned to reflect original data structures. The coloring denotes groups with similar LFC depletion rates. (Bottom) Vertical scatter plot showing changes in the LFC depletion rate of NhoodGroup(s). **C:** Bar plots of cell type distribution in Neighborhood Groups 2 and 5. **D:** Ridge plot of *Nos2* gene expression across AM clusters, segmented by timepoint of infection. **E:** Volcano plot of Differential Gene Expression (DGE) in CD38^+/−^ AMs, with x-axis for log2 fold changes and y-axis for −log10 adjusted p-values, highlighting top DGE genes for both groups. **F:** Horizontal bar chart illustrating Gene Ontology (GO) Processes Enriched in CD38^−^ AMs. **G:** Bar chart illustrating the temporal distribution of infected AMs by CD38 staining, with each bar representing the percentage of the specific cell type within the total infected AMs at each timepoint. **H:** Horizontal bar chart illustrating the Gene Set Enrichment Analysis for CD38^+^ AMs.

Work by Rothchild et al. highlighted that murine AMs exhibit a robust anti-inflammatory response during the early stages of tuberculosis infection, up to 10 dpi. This early-phase response is postulated to be pivotal to their role in the initial infection process^2,6,8,10^. Building on this previous work, our focus on the AM populations revealed that the CD38^−^ AM subset dominates this early infection landscape. Specifically, they account for approximately 70% of the Mtb-infected host cells at 14 dpi (Figure 5A). To gain a more nuanced understanding of the CD38^−^ AMs and the impact of their changing numbers, we employed differential abundance testing (DAB) to group previously identified neighbors based on their log-fold change (LFC) depletion rates, as an alternative approach to the traditional cluster-based categorization (Figure 5B)^77^. Through this approach, we identified two neighbor groups (group 2 and 5) exhibiting significant depletion rates in late infection (> 5 LFC). Examining the cell types associated with these high LFC depletion rate groups (FDR <0.1), we found that 98% of cells from group 2 belonged to the CD38^−^ TR-AMs subsets (spanning CD38^−^ TR-AM_1, CD38^−^ TR-AM_2, CD38^−^ TR-AM_3). For group 5, 57% were associated with the CD38^−^ TR-AMs and 33% with CD38^+^ moAMs (Figure 5C). The over-representation of CD38^−^ TR-AMs in groups marked by pronounced depletion rates, identified through our DAB approach and independently confirmed by compositional analysis through scCODA^79^ (Supp. File 1) , suggests an innate susceptibility of these cells to Mtb infection. This vulnerability becomes more obvious when compared with the unchanging relative abundance of CD38^−^ TR-AMs in bystander populations across different infection timepoints (Figure 4A and 4D). Coupled with the observed decline in the overall count of infected AMs as the infection unfolds (Figure 4C), it becomes evident that very few CD38^−^ TR-AMs become infected in the later stages. This underscores the idea that the CD38^−^ TR-AM clusters, dominant at 14 dpi, are poorly equipped to survive Mtb infection. This hypothesis is supported by the absence of *Nos2* mapping reads across all infection timepoints in CD38^−^ TR-AMs (Figure 5D), excepting cells bordering the MoAM in the CD38^−^ TR-AM_1 cluster (likely signifying a common scRNA-seq occurrence of cells being misattributed among neighboring clusters).

DGE analysis of the CD38^−^ AMs further supports this hypothesis. We found CD38^−^ AMs to be associated with known markers of AM populations (*Chil3, Lpl, Marco, Mrc1, Trf*)^8,9^ (Figure 5E). Examining the transcriptional profile of these populations we observed an upregulation of genes involved with lipid metabolism such as *Fabp4, Mgll, and Lpl*^8,80^. Additionally, we noted increased expression in genes linked to the electron transport chain (like *mt-Nd1, mt-Nd2, mt-Nd4, mt-Cytb, and mt-Atp6*) which play pivotal roles in cellular energy metabolism. This metabolic transcriptional signature was further complemented by the upregulation of genes connected to fatty acid uptake and transport (e.g., *Dbi*)^81^, lipid droplet formation (e.g., *Cidec, Plin2*)^82,83^, and protection against oxidative stress (e.g., *Gpx1, Gpx4*)^84^ (Supp Table 2). Collectively, this data indicates a shift towards fatty acid oxidation (FAO) metabolism. Importantly, the upregulation of genes involved in metabolic and homeostatic functions stands in contrast to a diminished expression of pro-inflammatory genes, critical in combating tuberculosis infection (Figure 5E) (Supp. Table 2). Moreover, Gene Ontology (GO) functional enrichment analysis of the transcriptional profile of CD38^−^ AMs also supports these observations. The functional profile of the CD38^−^ TR-AMs aligns with alveolar macrophage characteristics that have been shown to promote Mtb growth^6^. These include increased oxidative phosphorylation and fatty acid metabolism (Figure 5F). In summary, our data suggests that the broad range of anti-inflammatory responses often associated with AM population are mediated through CD38^−^ TR-AMs, which are highly susceptible and sensitive to Mtb infection during the early phases of TB.

### CD38^+^ AMs are key contributors in controlling tuberculosis infection.

We re-clustered the AM subsets based on their surface expression of CD38 protein. Consistent with the previous observations, CD38^+^ cells only constituted 12% of the total infected AMs at 2 weeks, but this proportion increased to 89% and 93% at the 4 and 6-week timepoints, respectively (Figure 5G). Intriguingly, DAB testing revealed unchanging abundance of infected CD38^+^ AMs across timepoints (Figure 4A), and mo_AMs and CD38^+^ TR-AMs constitute the majority of the infected alveolar macrophages in the later stages of infection (Figure 4B–4C). This stability in numbers, even amidst a pronounced decline in the overall infected AMs (Figure 4C), implies that these cellular subsets might be inherently more resilient to Mtb infection. This interpretation aligns with their observed *Nos2* expression patterns. Both CD38^+^ TR-AMs and mo_AMs exhibit a bimodal *Nos2* expression, with increasing cell counts and *Nos2* expression intensity, suggesting their antimicrobial capacity increases over time (Figure 5D). The elevated CD38 expression and pro-inflammatory pathways in AMs in latter stages, as described earlier, are predominantly represented by these two CD38^+^ populations.

Trajectory and pseudotime analyses provide additional insights into these populations. While mo_AMs, CD38^−^ TR-AM_1, and CD38^−^ TR_AM_2 appear as mature endpoints (grey circles 12,18,17,7,5 and root 3, white circle), CD38^+^ TR-AMs seem to arise from enhanced polarization of the CD38^−^ TR-AM_3 subset (root 1, white circle and branching point 12,17, black circles, Figure 2B). Thus, we hypothesize that the decreased presence of the CD38^−^ TR-AM_3 subset in subsequent infection stages is likely a consequence of these cells transitioning to CD38^+^ TR-AMs, as highlighted in the subsequent paragraphs. Finally, enrichment analysis of the genes upregulated by CD38^+^ AMs reveals up-regulation of the same pathways that are associated with control of Mtb infection (Figure 5H). These findings suggest that the divergent behavior of CD38^+^ and CD38^−^ AMs in response to Mtb infection is programmed into the cell subsets prior to infection, with the CD38^+^ AMs playing a potentially crucial role in managing Mtb growth and spread.

### Intrapulmonary BCG vaccination amplifies the CD38^+^ macrophage population resulting in enhanced control of Mtb infection.

Recent investigations, including those by Mata et al. 2021^14^, have shown that intrapulmonary BCG vaccination prior to Mtb infection induces protective responses in lung resident macrophages. To extend this observation, we assessed how pulmonary BCG administration modulates the responses of CD38^+/−^ macrophages to Mtb infection. Mice were vaccinated intranasally with live BCG and after a two-month period, were infected with the reporter *smyc’*::mCherry/*hspx’*::GFP Mtb Erdman for a duration of two weeks. Subsequently, we integrated scRNA-seq datasets from these vaccinated mice with our existing timepoint analysis data.

**Figure 6:**
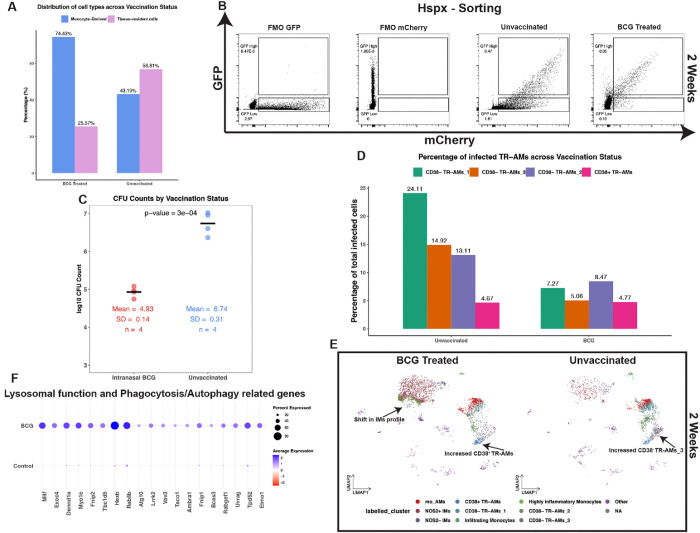
Comparative Analysis of Immune Responses, Cellular Distribution, and Mycobacterium tuberculosis Infection Dynamics in BCG-Vaccinated vs. Unvaccinated Mice. **A:** Bar chart comparing the proportions of Monocyte-Derived and Tissue-resident cells between BCG-vaccinated and unvaccinated mice at 2 weeks post-infection. N= 3931 cells (5 mice) for the BCG vaccinated group and n=2278 cells (5 mice) for the Unvaccinated Group. **B:** Flow cytometry analysis of the distribution of the Mtb mCherry and GFP signals from infected cells in BCG-vaccinated and control mice. **C:** Scatter plot showing log10-transformed CFU counts in BCG-vaccinated and unvaccinated mice at 2wpi. Points on the plot represent individual CFU counts, with horizontal bars illustrating the mean values for each group. Summary statistics including mean CFU count, standard deviation (SD), and sample size (n) are provided in the plot. A p-value annotation derived from a t-test comparing the differences in mean between the two groups is included in the upper part of the plot. The y-axis is labeled with “log10 CFU Count” to reflect the log transformation. **D:** Bar chart displaying the distribution of infected TR-AM subtypes by vaccination status. Each bar’s height corresponds to the percentage of the respective TR-AM subtype within the total infected macrophage cell population, with values labeled on each bar. **E:** UMAP visualization of cell clusters in BCG vaccinated and control samples, 2 weeks post-infection. N= 3931 cells for the BCG vaccinated group and n=2278 cells for the Unvaccinated Group. Both groups include cells pooled from 5 different animals. **F:** Dot plot representation of genes associated with lysosomal function and phagocytosis/autophagy pathways, between BCG-vaccinated and control mice. Dot size and color intensity indicate percentage and average expression level, respectively.

Analysis of infected macrophage populations from both BCG-vaccinated (n=3931 cells) and unvaccinated (n=2278 cells) mouse lungs, two weeks post-Mtb infection, revealed marked differences. In the BCG-vaccinated cohort, the monocyte-derived macrophages, associated with *hspx’*::GFP-high Mtb (Figures 1F and 2E), constituted the vast majority of infected macrophages, at 74.43%. This is in stark contrast to the unvaccinated cohort, where such cells accounted for just 43.19% (Figure 6A). Flow cytometry data from the sorted populations reinforced these findings. Macrophages from the BCG-treated group mounted a more effective response, limiting Mtb replication more efficiently. This is underscored by a marked reduction of the mCherry signal in infected cells of BCG-vaccinated mice, which was approximately ~2 log_10_ times lower than that in infected cells from unvaccinated mice. Moreover, there was a substantial difference in the proportion of *hspx’*::GFPstressed bacteria between the two cohorts: GFP-high cells represented 22.6% of the total infected cells in the unvaccinated group compared to 77.78% in their BCG-vaccinated counterparts (Figure 6B). The distribution of *hspx’*::GFP^+^ cells between the two groups, as revealed in our scRNA-seq dataset (Supp. Figure 4B), aligned with our flow cytometry data. Complementing these findings, we observed a marked decrease in the overall percentage of infected cells in BCG treated mice (0.45%) compared to unvaccinated mice (2.08%) (Figure 6B), as also confirmed by independent flow cytometry analysis (Supp Figure 4A). This was also supported by an overall reduction in bacterial burden in BCG-treated mice, as assessed by CFU counts (Figure 6C). These differences in cellular responses between BCG-treated and unvaccinated mice highlight significant alterations in their macrophage populations. BCG-treated mouse lungs were dominated by highly pro-inflammatory monocytes, and there was a marked reduction in infected CD38^−^ TR-AMs compared to unvaccinated controls (Figure 6D and 6E). The reduced proportion of infected CD38^−^ TR-AMs in BCG-treated mice is likely the result of increased recruitment of pro-inflammatory monocytes, in addition to the transition of the CD38^−^ TR-AMs_3 into their CD38^+^ counterparts (Figure 6E).

Using WGCNA, we identified a unique co-expression module of 463 genes exclusive to the macrophage populations of BCG-vaccinated mice (Supp. Figure 4C) (Supp. Table 3). This module was highly enriched in pro-inflammatory genes, including *Nos2* and *Cd38* (Supp Figure 4D). Further analysis revealed significant presence of genes involved in small GTPase signal transduction, encompassing Rab GTPases, guanine nucleotide exchange factors (GEFs) and GTPase activators (GAPs) (Supp. Figure 4E). Rab GTPases are known for their roles in endosomal trafficking, while GAPs and GEFs are vital for membrane transport, phagocytosis, and controlling of the actin cytoskeleton^85–87^. Their increased expression suggests modifications in intracellular trafficking within the BCG-treated macrophages. Altered Mtb intracellular trafficking and increased lysosomal fusion can limit bacterial growth ^88–90^ and amplify killing mechanisms due to immune activation and increased autophagy^91–95^. Our data confirms the previous findings as we observe that increased intracellular trafficking is tightly linked to increased expression of genes involved in lysosomal and autophagy functions in BCG-treated macrophages (Figure 6F). Additionally, genes facilitating macrophage migration and tissue invasion saw heightened expression in macrophages from BCG-treated lungs (Supp.Table 3). This observation aligns with the gene-set enrichment analysis of the 463 co-expressed genes from the scWGCNA module. Here, cell migration, motility, programmed cell death and autophagy emerged as the pathways enriched in BCG-treated macrophages, in both GO and KEGG analysis (Supp Figure 5A) (Supp Table 4).

The increased expression of pro-inflammatory gene signatures involved in control of Mtb infection in BCG-treated macrophages aligns with their observed phenotype. A hallmark feature across BCG-treated mice was the increased expression of CD38 (Supp. Figure 5B). This immune control phenotype was still evident at 4 weeks post-infection, corroborating Mata et al.’s findings (Supp Figure 5C). Intriguingly, we observed discrepancies between the two studies regarding the origin of infected macrophages after BCG vaccination. In our study, the majority of infected macrophages in BCG-treated mice were monocyte-derived, while previous data indicated Mtb was mostly confined to AMs after BCG vaccination^14^. We believe the disparity was due to weak CD64 staining on pro-inflammatory monocyte-derived cells (Supp Figure 5D). To test this hypothesis, we re-infected BCG-treated and control mice with a high infection dose (5×10^3^ CFU), similarly to Mata et al.^14^ The published CD64 flow cytometry gating approach will identify most infected macrophages as AMs in BCG-treated mice^14^ (Supp Figure 5E). However, when gating only on SiglecF, expressed uniquely by AMs (Figure 1B), the results indicate the majority of infected cells are monocyte-derived (Supp Figure 5F), as in our scRNA-seq dataset. Regardless of the gating method, we found a consistent increase in the percentage of infected AMs in BCG-vaccinated mice compared to the unvaccinated counterparts, underscoring their improved ability to restrict Mtb growth, both at 2wpi (Supp Figure 5F) and at 4wpi (Supp figure 5G)^14^. This aligns with our scRNA-seq results and highlights the fundamental role of the increased polarization of the CD38^−^ TR-AMs_3 transitioning to CD38^+^ TR-AMs for enhanced Mtb control in BCG-treated AMs (Figure 6E). In conclusion, our data suggests that the heightened defense against Mtb reinfection observed after intranasal BCG vaccination is due to both the increased presence of CD38^+^ monocyte-derived macrophages and the activation of resident CD38^+^ TR-AMs.

### Pre-existing differences in the chromatin organization of CD38^+^ vs CD38^−^ AMs are linked to differential responses to Mtb infection.

Our scRNA-seq analysis revealed distinct differences in AM populations between naïve and Mtb-infected mice. Specifically, we observed that CD38^+^ moAMs are recruited to the lung in response to Mtb infection (Figure 2A). In contrast, CD38^+^ TR-AMs were already present prior to infection, predominantly exhibiting a less active CD38^−^ phenotype, which we previously defined as CD38^−^ TR-AM_3 (Figure 2A and 2B).

**Figure 7:**
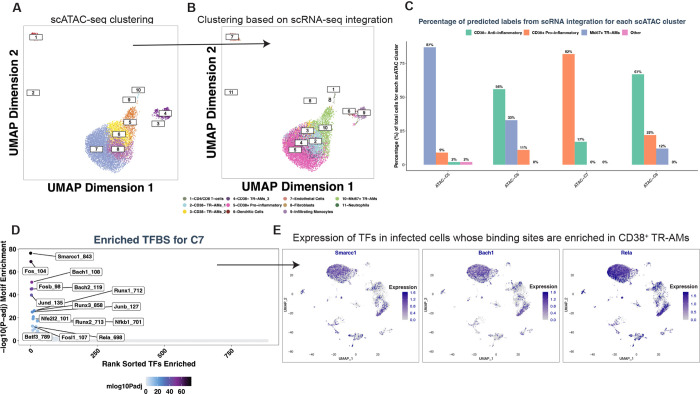
Pre-existing differences in the chromatin organization of TR-AMs are linked to differential responses to Mtb infection. **A:** Unbiased UMAP Clustering of Chromatin Accessibility Profiles. Cells are spatially organized and colored based on similarities in their chromatin accessibility patterns. Associated cluster names are shown on the plot. **B:** Visualization of unconstrained integration of single-cell ATAC-seq and scRNA-seq datasets, with each point being an individual cell spatially organized by chromatin accessibility profile and colored according to its predicted cell type, based on cell types in the scRNA-seq dataset. **C:** scATAC Cluster Compositions Post-scRNA Integration. This bar plot depicts the percentage compositions of each scATAC cluster based on the predicted cell labels obtained after integration with the scRNA dataset. The x-axis represents different scATAC clusters, while the y-axis displays the percentage of total cells for each scATAC-seq cluster associated with each predicted scRNA-seq cell label. Each bar is colored distinctly for the three TR-AMs conditions: “CD38^−^ Anti-inflammatory,” “CD38^+^ Pro-inflammatory,” “Mki67^+^ TR-AMs,” and “Other.” The actual percentage values are overlaid on the bars. **D:** Scatter plot depicting TFBS enrichment scores in cluster C7, with each point representing a transcription factor (TF) ranked on the x-axis and plotted against the negative log10 of its adjusted p-value on the y-axis. Top transcription factors are labeled based on their enrichment scores. **E:** UMAP plots displaying log-normalized count expression levels for the transcription factors ‘*Smarcc1’*, *‘Bach1’*, and *‘Rela’* in the infected cell population of the scRNA-seq dataset.

To investigate the chromatin landscape and potential epigenetic regulation of AM subsets prior to infection, we performed scATAC-seq on CD45^+^ cells isolated from the lungs of naïve mice. Unbiased clustering based on differential chromatin accessibility identified 10 distinct clusters (Figure 7A). Integrating this scATAC-seq dataset with our timepoint-specific scRNA-seq data, which includes naïve, bystander, and infected cells, revealed that variations in chromatin organization before infection align closely with the diverse transcriptional phenotypes observed during tuberculosis infection. Using gene scores, we inferred the potential gene expression profiles for each cell in the scATAC-seq sample, based on the accessibility of regulatory elements adjacent to each gene. We then performed data integration with the scRNA-seq dataset, as previously described^96^ (Figure 7B).

Given that our scATAC-seq sample comprised only cells from naïve mice, we anticipated that the inferred gene expression profiles from the scATAC-seq dataset would predominantly align with those of naïve cells from our scRNA-seq dataset. Surprisingly, we found that the inferred gene expression of cells from cluster C7 mirrored that of the pro-inflammatory CD38^+^ subsets from our scRNA-seq data (Figure 7B and 7C), which by transcriptional profiling are not present in naïve mice (Figure 2A). In contrast, clusters C6 and C8 aligned with CD38^−^ TR-AMs, while cluster C5 correlated with Mki67^+^ AMs (Figures 7B and 7C). To validate that cluster C7 represented CD38^+^ TR-AMs and not monocyte-derived AMs, we probed for open chromatin within the promoter regions of monocyte markers *Mafb* and *Ly6a*, whose expression is restricted to monocyte-derived macrophages in our scRNA-seq dataset, as noted earlier (Figure 1F). We found high levels of open chromatin for these markers only in monocyte-derived cells, but not in cluster C7 (Supp. Figure 6B).

To further understand why the inferred gene expression of cluster C7 aligns with pro-inflammatory populations in scRNA-seq, we first assessed transcription factor dynamics, performing marker peak and motif enrichment analysis (FDR < 0.1, log2FC > 0.5) to identify transcription factor binding sites (TFBS) that are enriched across the different scATAC-seq clusters. Cluster C7 exhibited highly significant enrichment for binding sites of transcription factors such as *Smarcc1, Bach1*, and *Rela*, known to drive pro-inflammatory gene activation in macrophages^97–99^ (Figure 7D). We further validated the expression of these TFs in our scRNA-seq dataset and found them to be uniquely expressed by CD38^+^ pro-inflammatory macrophages following Mtb infection (Figure 7E).

Finally, our analysis of open chromatin peaks within the regulatory regions (± 5k from TSS) of pro-inflammatory genes that define CD38^+^ AMs, such as *Nos2, Cd38, Slc7a11, Ccl5, Ptgs2, Il1b, and Cxcl2*, revealed higher open chromatin in cells from cluster C7 compared to clusters C6 and C8, further validating that CD38^+^ TR-AMs are pre-primed for a pro-inflammatory response (Supp Figure 6C).

Overall, our analysis demonstrates that CD38^+^ TR-AMs are present in naïve mouse lungs before infection, but are transcriptionally quiescent and have an epigenetic profile markedly distinct from their CD38^−^ TR-AM counterparts. These CD38^+^ TR-AMs have increased chromatin accessibility in promoter regions of pro-inflammatory genes, with significant enrichment of transcription factor binding sites that have the potential to drive pro-inflammatory macrophage activity and control Mtb growth. Our findings support the hypothesis that the response of different TR-AM subsets to Mtb infection is largely predetermined by their intrinsic chromatin organization prior to infection.

## Discussion

Our understanding of immune protection against tuberculosis largely comes from studying immune failure^31,100^. Experimental infections in immune deficient mouse strains have informed us which pathways, when compromised, increase susceptibility to infection and disease^101^. Similarly, several mutations in the human population have been linked to increased incidence or severity of disease^102,103^. However, if disease outcome is determined by the biology of the host macrophages, and if different macrophage populations are responsible for control or promotion of bacterial growth, focusing solely on immune failure offers a limited view of disease control. This focus has resulted in our reliance on Interferon-g Release Assays (IGRA), Mycobacterial Growth Inhibition Assays (MGIA) and similar biological readouts for vaccine development, which have proven to be non-predictive of immune protection^27,28^.

Our current study uses a mouse challenge model with fluorescent fitness reporter bacteria to define and characterize different macrophage populations in the infected mouse lung. We’ve previously found that recruited pro-inflammatory monocyte-derived IMs effectively control Mtb,^6,8,9^ while AMs exhibit diverse phenotypes. We posit that the variability among these macrophage subsets significantly influences disease progression in tuberculosis.

In early infection, Mtb primarily resides in CD38^−^ AMs, which show a muted response, as noted by Rothchild et al^10^. As infection progresses, the AM landscape changes significantly with the recruitment of moAMs and activation of a subset of TR-AMs, leading to increased bacterial control. These macrophage subsets transition the lung environment from immune homeostasis to a pro-inflammatory state, effectively curtailing Mtb growth. The shifts in macrophage phenotypes are rooted in their intrinsic epigenetic programming, as revealed by ScATAC-seq analyses. CD38^+^ TR-AMs, are already present in naïve lungs, exhibit chromatin landscapes predisposed for a pro-inflammatory response, indicating epigenetic priming as a key factor in their infection response. The diverse chromatin organization of the different TR-AMs subsets before infection suggests the potential for manipulating their epigenetic programs, opening new avenues to enhance macrophage function in TB.

Furthermore, examination of post-infection phenotypic changes in AM subsets, particularly after intrapulmonary BCG administration, provides crucial insights into potential strategies for reprogramming these macrophages to our advantage. Our focus was not on promoting BCG as a long-term vaccination strategy, but rather on understanding how transient changes in macrophage function post-BCG administration can inform potential therapeutic targets. We observed transcriptional shifts resulting in the polarization of CD38^−^ TR-AM_3 towards the CD38^+^ TR-AM phenotype and increased inflammatory activation of monocyte-derived macrophages in BCG-treated mice. These transcriptional responses are associated with augmented expression of pathways related to intracellular trafficking and lysosomal/autophagic functions, suggesting that intranasal BCG administration triggers the pre-activation of genetic programs inherent to the epigenetic profile of pro-inflammatory TR-AM subsets. This promotes a phenotype more effective in restricting Mtb replication. Additionally, BCG also increases the recruitment of monocyte-derived macrophages, resulting in fewer unresponsive CD38^−^ TR-AMs being infected with Mtb, further strengthening the lung myeloid populations’ ability to counter the Mtb challenge. These results extend the recent reports that BCG reprograms lung AMs to better control Mtb^14,17^. While our findings, conducted in SPF mice, do not fully capture the complex genetic and environmental influences found in human populations, the changes in macrophage phenotype observed during infection and post-BCG vaccination offer a clear path towards improved tuberculosis control. We propose that the dynamic nature of these macrophage subpopulations plays a major role in the early events following Mtb infection and throughout the course of the disease. Through understanding the underlying molecular mechanisms and the broader immune context driving the distinct responses of CD38^+^ and CD38^−^ AMs to Mtb infection, we could guide the screening of therapeutics aimed at improving macrophage control of Mtb early in infection. However, factors such as genetic diversity, nutritional status, co-infections, and early-life BCG vaccination can significantly alter immune responses; therefore, these results will require validation under more complex conditions in humans.

The significant shifts in macrophage phenotypes we’ve observed, especially in the context of BCG vaccination and immune function, emphasize the functional resolution of the analytical tools employed in this current study. The WGCNA method, a focal point of this and ongoing studies, has produced gene expression modules that functionally categorize various macrophage subpopulations, in both mouse and NHP infections. These modules, alongside newly identified CD markers, facilitate integration with skin and lung challenge approaches for more accurate phenotype identification of tissue-resident and monocyte-derived macrophages in relation to disease or vaccination status. We believe this represents a viable avenue to the development of predictive biomarkers for immune protection.

## MATERIALS AND METHODS

### Mtb and BCG strains

The parental strain employed for all experiments was *Mycobacterium tuberculosis* Erdman (ATCC 35801). Fluorescent reporter strains including smyc′::mCherry, smyc′::mCherry/*hspx’*::GFP, and hsp60′::GFP have been reported^35,104–106^. Both the *M. tuberculosis* strain and BCG (Pasteur) were cultivated at 37°C until they reached the mid-log phase in MiddleBrook 7H9 broth enriched with 10% OADC (Becton, Dickinson and Company), 0.2% glycerol, and 0.05% tyloxapol (Sigma-Aldrich). For the selection of fluorescent strains, Hygromycin B (50 mg/ml) was utilized. For mouse infections, bacterial aliquots were prepared in 10% glycerol, titrated, and preserved at − 80°C, following the protocol detailed in Pisu et al., 2023^18^.

### Mice

C57BL/6J WT, B6.129P2-Nos2tm1Lau/J (NOS2^−/−^), B6.129S7-Ifngtm1Ts/J (IFNγ^−/−^), B6.129S7-Rag1tm1Mom/J (Rag1^−/−)^ mice were purchased from The Jackson Laboratory. The mice used in this study were 6–8 wk old. All mice were maintained in a specific pathogen–free animal biosafety level 3 facility at Cornell University. Animal care was in accordance with the guidelines of the Association for Assessment and Accreditation of Laboratory Animal Care. All animal procedures were approved by the Institutional Animal Care and Use Committee of Cornell University.

### Mice infection and lung cells isolation

For *Mtb* infections, mice were anesthetized and intranasally inoculated with 1.5X10^3^ CFUs of the Erdman strains (*smyc′*::mCherry, *hspx′*::GFP/*smyc′*::mCherry, or *hsp60′*::GFP) resuspended in 30 μl of PBS containing 0.05% Tween 80. The inoculum dose was verified by plating various dilutions of the bacterial stocks used for infection on 7H10 agar plates supplemented with OADC Enrichment and glycerol. These plates were incubated at 37°C, and after 3 weeks, colonies were counted. At 2, 3, 4, and 6 weeks post-infection (w.p.i.), mice were euthanized. Lungs were aseptically removed and immersed in PBS containing 5% FBS and Collagenase IV (250U/mL). To preserve the gene expression profiles of both the host and bacteria, samples were immediately processed using a GentleMACS tissue dissociator (Miltenyi Biotec) and maintained on ice. The dissociated lung material was subsequently strained through a 70-μM mesh, and red blood cells were lysed using ammonium-chloride-potassium (ACK) lysis buffer (Lonza)^18^.

For BCG vaccinations, mice received an intranasal dose of 2×10^6^ CFU of BCG (Pasteur) bacilli in 30 μL of PBS containing 0.05% Tween 80. Post-infection, these mice were housed in pathogen-free cages at a biosafety level 2 facility at Cornell University, in preparation for re-infection with *M. tuberculosis* Erdman. After a period of two months (60 days) from the initial vaccination, these mice underwent a secondary intranasal challenge. This challenge involved approximately 1.5×10^3^ or 5×10^3^ CFU of either *hspx′*::GFP/*smyc′*::mCherry or *smyc′*::mCherry Mtb Erdman respectively, with the bacteria also resuspended in 30 μL of PBS containing 0.05% Tween 80. At specified intervals post-infection, specifically at 2 and 4 weeks, the lungs of these mice were aseptically removed and immersed in a solution of PBS containing 5% FBS and 250U/mL of Collagenase IV. The harvested lung tissues were then processed for subsequent scRNA-seq or flow cytometry analyses.

Sorting of the murine lung suspensions for scRNA-seq analysis

### Infected populations

To generate single cell suspensions for cell sorting, we followed the steps 4–18 of the previously published protocol^107^. In brief, cells from infected mice (n = 5/timepoint) were washed in PBS containing 5% FBS, resuspended in sorting buffer (PBS, 1% FBS, 5 mM EDTA, and 25 mM Hepes), filtered through a 40-μM strainer, and sorted. Throughout the sorting, samples were kept at 4°C and directly collected into Cell Staining Buffer (BioLegend). Mice infected with either smyc′::mCherry or *hsp60′*::GFP were used as a control to define the sorting gates for the *hspx′*::GFP/*smyc′*::mCherry-infected cells. For the BCG analysis, we followed the same protocol sorting n = 5 mice.

### Bystander populations

Single-cell suspensions from mice infected with smyc′::mCherry (n = 3 / timepoint) were incubated for 20 min with fluorophore-bound CD45 antibodies (104; BD). After two PBS washes, samples were resuspended in the sorting buffer, filtered via a 40-μM strainer, and sorted. During sorting, samples were consistently held at 4°C and collected directly into Cell Staining Buffer (BioLegend).

scRNA-seq libraries preparation and sequencing

### Sample Preparation and Staining

Sorted cells were centrifuged at 500 g for 5 min and then resuspended in 50 μl of cell staining buffer containing 0.25 μg of TruStain FcX PLUS (BioLegend), followed by a 10 min incubation at 4°C. An ADT plus HTO antibody cocktail mix (50 μl) was then added to the samples, and the cells were further incubated for 30 min at 4°C. After two washes in cell staining buffer, differentially tagged samples (e.g., *hspx′*::GFPhigh/*hspx′*::GFPlow) were combined and resuspended in 1× Dulbecco’s PBS. The samples were then fixed by slowly adding ice-cold methanol to a final concentration of 90% (vol/vol) and stored at − 20°C overnight.

### Sample Rehydration and mRNA Library Preparation

Post-fixation, the samples were brought out of the BSL3 facility, equilibrated on ice for 15 min, and washed twice with rehydration buffer (1× Dulbecco’s PBS with 1.0% BSA [Thermo Fisher Scientific] and U/μl RNase Inhibitor [Sigma-Aldrich]). The cell count was determined prior to loading onto the 10× chip. For mRNA library preparation, we adapted the 10× protocol (CG000206 Rev D), making a minor alteration in step 2.2. Specifically, we incorporated 1 μl of ADT and HTO additive primers (0.2 μM stock) following the method described by Stoeckius et al^108^. HTO and ADT libraries were prepared according to BioLegend’s standard protocols.

### Library Generation and Sequencing

The mRNA, HTO, and ADT libraries underwent quality control assessment using an Agilent Fragment Analyzer. Their concentrations were determined using the QX200 digital PCR system from Bio-Rad. Libraries were pooled in the same sequencing run at specific ratios: 90% mRNA, 5% ADT, and 5% HTO. Sequencing was performed on the NextSeq2000 (Illumina) using the 50-bp P3 NextSeq kit. The cycle distribution was: read 1 (28 cycles), i7 index (8 cycles), and read 2 (52 cycles). Sequencing depth exceeded 50,000 reads/cell.

#### scATAC-seq nuclei isolation, library preparation and sequencing

Murine naïve lung sorting was performed as described in Pisu et al^9,18^. For nuclei isolation, sorted cells were centrifuged at 300 rcf for 5 minutes at 4°C and then resuspended in 150uL of PBS supplemented with 0.04% BSA. A 100μL aliquot of this cell suspension was transferred to a 0.2mL flat-cap tube, centrifuged again under the same conditions, and subsequently resuspended in 50uL of Lysis Buffer (containing 10mM Tris-HCL, 10mM NaCl, 3mM MgCl2, 0.1% Tween 20, 0.1% Nonidet P40 Substitute, 0.01% Digitonin, and 1% BSA). This was incubated on ice for 4 minutes. Post-incubation, 50μL of Wash Buffer (comprising 10mM Tris-HCL, 10mM NaCl, 3mM MgCl2, 1% BSA, and 0.1% Tween 20) was added to the lysed cells. The nuclei suspension was then centrifuged at 500 rcf for 5 minutes at 4°C. The nuclei were washed with 45μL of a 1:20 dilution of the Nuclei Buffer (10x Genomics, PN-2000153/2000207) and centrifuged again using the same conditions. Finally, the isolated nuclei were resuspended in a volume of Diluted Nuclei Buffer to obtain a concentration ranging from 3080 to 7700 nuclei/μL. This was used as input for the 10X protocol (CG 000209 Rev D) targeting a recovery of 10,000 nuclei.

The transposition reaction and library construction were performed following the protocol from 10X (CG000209 REV D). Sequencing was conducted on a NextSeq 500 with the parameters: Read 1N: 50 cycles, i7 index: 8 cycles, i5 index: 16 cycles, and Read 2N: 50 cycles.

#### Antibodies Used for scRNA-seq

For our scRNA-seq timepoint experiments, we used a range of TotalSeq (BioLegend) murine antibodies in our antibody cocktail mix, each at a concentration of 0.5 μg/sample. These antibodies included SiglecF (custom-made, clone S17007L), CD64 (cat. # 139325), Ly6G (cat. # 127655), CD11c (cat. # 117355), CD14 (cat. # 123333), Ly6G-Ly6C (cat. # 108459), CD63 (cat. # 143915), F4/80 (cat. # 123153), CD38 (cat. # 102733), TLR4 (cat. # 117614), CD11b (cat. # 101265), CD16/32 (cat. # 101343), CD86 (cat. # 105047), CD1d (cat. # 123529), CD3 (cat. # 100251), CD4 (cat. # 100569), and CD8a (cat. # 100773). In addition, for hashing purposes, we used BioLegend’s Hashtag 1 murine (cat. # 155801), Hashtag 2 murine (cat. # 155803) antibodies.

#### Flow cytometry analysis

Lung cell suspensions were counted and incubated for 30 min in the dark at room temperature with fluorophore-conjugated antibodies, washed twice with PBS 1×, and fixed in 4% paraformaldehyde. Antibody panels and Fluorescence Minus One controls were generated as appropriate. For this study, we used fluorochrome-conjugated mAbs specific to mouse SiglecF (E50–2440; Becton Dickinson), CD64 (X54–5/7.1; BioLegend), MerTK (2B10C42; BioLegend), CD38 (90; Biolegend) and CD45 (104; Becton Dickinson), along with the following reporter strains: smyc′::mCherry (mCherry), hsp60′::GFP (GFP), and hspx′::GFP/smyc′::mCherry. Cells were analyzed with a Symphony A3 (BD Biosciences). Data were analyzed using FlowJo software (version 10.9; BD).

#### Quantification of bacterial loads

At 2 weeks post-infection (for BCG-vaccinated) and at 4 weeks post-infection (for B6.129P2-Nos2tm1Lau/J (NOS2^−/−^)) mice were sacrificed and the lung lobes homogenized in PBS containing 0.05% tyloxapol (Sigma-Aldrich). Bacterial loads were determined by plating serial dilutions of the homogenates on 7H10 agar. Plates were incubated at 37°C and colonies enumerated 3–4 weeks after.

### Data Analysis

#### Data Acquisition and QC

Sequencing data derived from each run underwent processing using distinct software tailored to the library type. mRNA libraries were processed using the CellRanger software (v. 6.0) from 10X Genomics and the mouse mm10 genome (GRCm38). The ADT and HTO libraries were processed using CITE-Seq-Count (v. 1.4.3), available at https://hoohm.github.io/CITE-seq-Count/. This processing yielded raw count matrices for both mRNA and proteins. Downstream data analysis was conducted in Seurat (v. 4.3), following methods outlined by Stuart et al^109^. Cells with less than 200 unique genes or with over 30% of the total reads belonging to mitochondria were filtered out. The HTOs multiplexed samples were then demultiplexed using the MULTIseqDemux() function in Seurat^110^. This step allowed for the removal of doublets and empty droplets and ensured the correct assignment of identities to each sample.

#### Single-Cell RNA Sequencing Data Preprocessing:

All scRNA-seq datasets from individual timepoints were preprocessed using Seurat’s regularized negative binomial regression, where both the number of counts and the percentage of mitochondrial reads were regressed out, as per Hafemeister and Satija^111^. For analysis of the macrophage populations in the uninfected naïve lungs we used the previously generated datasets from Pisu et al^9^. Metadata columns, namely *“Timepoint”, “Batch”, “Infection Status”*, and *“Vaccination Status”*, were added to each dataset prior to subsequent steps.

#### Data Merging and Integration:

Datasets were combined into a unified Seurat object. The raw counts contained in the RNA slot of the merged object were used as an input for Harmony integration^112^. Raw counts underwent log-normalization, followed by the identification of the top 3,000 variable genes. These genes were scaled and centered. PCA was then performed on these values. Integration in Harmony incorporated *“Batch”, “Infection Status”, “Timepoint”*, and *“Vaccination Status”* as covariates.

#### Cluster Detection and Annotation:

Using the Harmony-aligned embeddings, graph-based cluster detection was achieved utilizing principal components (PCs) that had been marked statistically significant by the jackstraw method^113^. For community detection, we employed the Louvain algorithm^114^. Cell types for each identified cluster were annotated using both reference-based^115^ and canonical marker genes.

#### Trajectory and Pseudotime Analysis:

This analysis was carried out in Monocle (v3.0)^116^. The integrated Seurat object was converted using the SeuratWrappers package in R (v. 0.2.0). For unbiased trajectory and pseudotime analysis of the macrophage populations, all cells classified as macrophages were assigned to the same partition, and trajectory/pseudotime analysis was conducted as previously described^117^. White circles indicate the root origins of the trajectory, grey circles indicate destination fates and black circles indicate branching points.

#### Generation of a force layout embedding (FLE)

To visualize infected and bystander cells in a force layout embedding (FLE) we used the methods described in Waddington-OT^76^. Briefly, using the Pegasus library (pegasus), a PCA was performed on the expression data using the function pg.pca(). This was followed by the determination of nearest neighbors using pg.neighbors(). Subsequently, a diffusion map was generated through the function pg.diffmap() to visualize the transition between cellular states. To visualize the data, the FLE algorithm implemented in Pegasus was employed by invoking pg.fle(). The resulting 2D coordinates were then extracted for further visualization. A scatter plot was generated using matplotlib to represent cells in the 2D space derived from the FLE calculations. Cells were color-coded based on their respective timepoints (weeks).

#### Time-dependent pathway analysis

For our time-dependent pathway analysis, we employed the Tempora package^74^. First, the integrated Seurat object was subset to only contain macrophage clusters. Subsequently, the “ImportSeuratObject()” function was employed to prepare the data for Tempora, where clusters and timepoints were explicitly defined. Given the necessity of pathway enrichment analysis, gene set files were fetched from the BaderLab online resource. We specifically selected gene set files that incorporated all pathways, excluding those inferred from electronic annotations (IEA). The downloaded gene matrix transposed (GMT) file was then leveraged for pathway enrichment calculations using the GSVA method with “CalculatePWProfiles()”. To construct the cellular trajectory, the “BuildTrajectory()” function was applied, with the number of principal components set to 11 and a statistical significance threshold of < 0.05. The trajectory visualization was achieved using the “PlotTrajectory()” function, and subsequently, generalized additive models (GAMs) were employed to identify significant time-varying pathways through the “IdentifyVaryingPWs()” function. The temporal dynamics of these pathways were illustrated using a custom ggplot function, “ggplotVaryingPWs()” for improved visualizations.

#### WGCNA co-expression analysis

WGCNA is an analytical pipeline used extensively by developmental biologists, that support the iterative, unbiased assembly of gene expression modules that define cell populations of interest. To perform weighted gene co-expression network analysis on our scRNA-seq datasets, we employed the scWGCNA package^45^. Pseudocells were computed using the “calculate.pseudocells()” function from the scWGCNA package. This method aggregates single cells into pseudocells to reduce the complexity of the dataset. A fraction of 0.2 of the cells were used as seeds, and for each seed, 10 nearest neighbors were aggregated based on the Harmony dimensional reduction. For each analysis, the selected single cell data was normalized using the “LogNormalize()” method with a scaling factor of 10,000. Variable features were identified using the Variance Stabilizing Transformation (VST) method, and the top 3,000 features were retained.

Subsequently, pseudocells (pcells) and the variable genes (var.genes) identified in the previous step were used in the “scWGNA()” function to identify modules of co-expressed genes. Membership tables were inspected to understand the genes belonging to each module, and average expressions of each co-expression module per cell were analyzed. Eigengenes for the co-expression modules were computed using the “scW.eigen()” function. This function collapses the expression profile of each module into a single representative profile known as the module eigengene. The average expression of each module was then visualized on a UMAP plot using a customized “scW.p.expression()” function for improved visualization.

#### Differential Abundance Testing with Milo

We used the neighborhood-based statistical framework “Mylo” ^77^ to test for changes in the abundance of infected and bystander macrophage populations across timepoints. To determine the best parameters for running the model both the neighborhood cell distribution and the distribution of uncorrected P-values were assessed. A k-nearest neighbors (k-NN) graph was constructed on the data using the “buildGraph()” function, with *k* set to 10, and using Harmony as the dimensionality reduction method. The neighborhoods were then defined on this graph using the “makeNhoods()” function, using the same *k* and *d* parameters as before. Subsequently, cells were counted within these neighborhoods based on their originating samples using the “countCells()” function. The neighborhoods were tested for differential abundance using the “testNhoods()” function. This test took into account a design matrix (consisting of a sample identifier and a variable for the timepoint) and the Harmony dimensions. Results were then sorted by the spatial False Discovery Rate (SpatialFDR). The neighborhood graph was further constructed using the “buildNhoodGraph()” function. Visualizations of the neighborhood graph highlighting the differential abundance results were then generated. Following this, neighborhoods were annotated based on cell identity, and a histogram was plotted to visualize the fraction of identified cells. Neighborhoods with an identity fraction less than 0.4 were labeled as “Mixed”. Bee swarm plots were generated to further visualize the data based on these identities. Finally, neighborhoods were grouped using the “groupNhoods()” function, with a max.lfc.delta of 2. The resultant grouped neighborhoods were visualized on a UMAP plot and further explored through bee swarm plots based on their LFC differences.

#### scCODA

We used scCODA to investigate changes in cell composition across different timepoints during Mtb infection. The cell count data was reshaped to match the format required for scCODA. This involved mapping the original timepoints to new categories (e.g., “2 Weeks” and “3 Weeks”‘ to “Early Timepoint”, “4 Weeks” and “6 Weeks” to “Late Timepoint”) and summing counts when necessary to consolidate the data into these new categories. The reshaped data was loaded into a pandas DataFrame, where each column represented a different timepoint and rows represented individual cell types. This DataFrame was then converted to an AnnData object, which is the data structure required by scCODA for compositional data analysis. The analysis focused on comparing the composition of macrophage subsets at these defined timepoints to identify significant changes in their proportions that could be linked to infection progression. The compositional analysis model was created using scCODA’s “CompositionalAnalysis” class, specifying “Timepoint” as the covariate and “automatic” as the reference cell type. The model was then run to perform Hamiltonian Monte Carlo (HMC) sampling and generating posterior distributions for the compositional changes between the specified groups.

Results from the scCODA model were summarized to determine which cell types showed significant changes in proportion relative to the reference group across the study’s timepoints.

#### Pathway enrichment analysis

Pathway enrichment analysis was performed using G::profiler^118^. For each analysis, we created an ordered by fold change (FC) (for DGE) or membership value (for scWGCNA modules) list of genes as a query, selecting only those genes where adjusted P value (p-adj) < 0.05. The analysis was performed using the g:SCS method for multiple testing correction, the gene ontology (GO), KEGG and Reactome databases as a data source, and the default settings for the other parameters in G::profiler. Only pathways enriched with p-value < 0.05 were considered statistically significant. Manual exploration of the gene lists for each analysis has also been performed to identify relevant themes for genes whose function is described in the literature (e.g., Small GTPase signal transduction). For this purpose, we only considered genes whose FC was absolute > 1.5 and p-adj < 0.05, or membership value for scWGCNA modules > 0.4.

#### scATAC-seq chromatin accessibility analysis

We utilized the ArchR software^96^ to perform integrative single-cell chromatin accessibility analysis. For the analysis we used the precompiled version of the mm10 genome in ArchR. Quality filtering parameters for data pre-processing included filtering out cells with TSS enrichment scores below 4 and less than 1000 unique fragments. As an additional step for quality control, we performed doublet identification and removal. Doublet scores were added to the Arrow files using the “addDoubletScores()” function with parameters *knnMethod* = UMAP, *k* = 10 and *LSIMethod* = 1. Post doublet identification, cells suspected to be doublets were filtered using “filterDoublets()”, reducing the initial nuclei count from 9371 to 8493. Subsequently, dimensionality reduction has been performed using the Iterative Latent Semantic Indexing (LSI) on the insertion count matrix with the “addIterativeLSI()” function in ArchR for 4 iterations. Clustering was performed on the IterativeLSI dimensions using Seurat with a resolution of 0.7. UMAP embeddings were added with the addUMAP() function using parameters *nNeighbors* = 30, *minDist* = 0.5 and *metric* = cosine. The UMAP was plotted and color-coded by clusters.

To identify marker genes for each cluster, gene expression for marker genes was estimated from chromatin accessibility data by using gene scores. A gene score is considered as a prediction of how highly expressed a gene will be based on the accessibility of regulatory elements in the vicinity of the gene^96^. To create the gene scores, we used distance-weighted accessibility models as defined in ArchR and implemented in the function “addGeneScoreMatrix()”. Marker genes were then identified for each cluster using the Gene Score Matrix with a Wilcoxon test method. The markers for each cluster were filtered using an FDR < = 0.05 and Log2FC > = 0.58. Imputed weights were added using the function “addImputeWeights()” to visualize the marker genes e.g., “*Mafb*” and *“Ly6a”* on the UMAP. To visualize the local chromatin accessibility around specific genes on a per cluster basis, the “plotBrowserTrack” function was employed, considering 5,000 base pairs both upstream and downstream of the start of the genes of interest.

#### Integration of scRNA-seq and scATAC-seq data

Single-cell timepoint RNA-seq data (scRNA-seq) was utilized for integration with ATAC-seq data. Unconstrained integration, a completely agnostic approach that takes all of the cells in the scATAC-seq experiment and attempt to align them to any of the cells in the scRNA-seq experiment, was used. The “addGeneIntegrationMatrix()” function was used to generate a gene integration matrix, which was named “GeneIntegrationMatrix”. For visualization, a palette was derived from the scRNA-seq data’s cell type categories. An embedding plot was generated using the “plotEmbedding()” function, colored by predicted cell groups to label and visualize the scATAC-seq clusters with the cell types predicted from our scRNA-seq dataset.

#### Transcription Factor (TF) Analysis

For TF analysis, the following steps were performed: 1) A reproducible peak set was determined using the “addReproduciblePeakSet()” function, with MACS2 as the peak caller^119^. The “getMarkerFeatures()” function was employed to identify marker peaks unique to an individual cluster or a small group of clusters in an unsupervised fashion, using the above calculated peak matrix and accounting for biases such as TSS Enrichment and fragment counts. Significant marker peaks were determined with criteria set at FDR < = 0.05 & Log2FC > = 0.58. 2) To assess whether marker peaks or differential peaks were enriched for binding sites of specific transcription factors, we performed motif and feature enrichment analysis. We annotated the ArchR project with motif information using the “addMotifAnnotations()” function, employing the “cisbp” motif set. Analysis of enrichment of motifs within marker peaks was performed with the “peakAnnoEnrichment()” function, using the following criteria FDR < = 0.1 & Log2FC > = 0.5. The enriched transcription factor binding sites (TFBS) for each scATAC-seq cluster were then visualized in a ranked scatter plot.

### Statistical Analysis

#### scRNA-seq

Differential expression analysis was performed using the nonparametric Wilcoxon rank-sum test as implemented in Seurat^109^. Only genes with FDR < 0.05 between two comparisons were considered statistically significant. Unless otherwise specified, the Wilcoxon rank-sum test followed by FDR correction has also been used to compare the distribution of a specific gene expression among two groups of cells in different plots and visualizations. For plots where the distribution of values (eg: CD38 protein level) have been compared, for each timepoint, across two conditions (eg: Bystander vs Infected) pairwise t-tests has been performed to determine which pairs of groups are different from each other. The p-values from the pairwise t-tests are adjusted for multiple comparisons using the Benjamini-Hochberg (BH) method. The Kruskal-Wallis test has been used to analyze the differences among group medians in a sample (eg: if the medians of protein expression levels differ significantly across clusters). The Dunn’s test has been used following the Kruskal-Wallis test to determine which specific groups’ distributions differ from each other and the Bonferroni method has been used to adjust p-values for multiple comparisons. In addition, effect sizes for differences in expression between groups were quantified using Cliff’s Delta, which measures the probability that a randomly selected value from one group will be greater than a randomly selected value from the other group. Cliff’s Delta values range from −1 to 1, where 0 indicates no effect, and values closer to −1 or 1 indicate stronger effects. The Cliff’s Delta was computed using the cliff.delta() function from the “effsize” R package. Data integration and batch effect removals were performed with Harmony as previously described^112^. ADT and HTO data were normalized using a centered log ratio transformation, implemented in the function “NormalizeData()” with normalization.method = ‘‘CLR,’’ in Seurat. RNA counts were log-normalized and scaled before PCA and data integration with Harmony. Data visualizations were generated on the log-normalized counts for the feature plots, scatter plots, and violin plots. Heatmaps and dot plot charts were generated on the scaled expression data, as per default in Seurat.

#### scATAC-seq

Marker genes distinguishing the different cell clusters were identified using the getMarkerFeatures() function on the GeneScoreMatrix. The Wilcoxon rank-sum test was utilized to assess the significance of predicted differential gene expression between cell clusters. Biases such as TSS Enrichment and fragment counts (log10(nFrags)) were accounted for during the analysis. Genes were considered as markers based on criteria set at a FDR of < = 0.05 and a Log2 Fold Change (Log2FC) of > = 0.58. The FDR was controlled using the Benjamini-Hochberg procedure. To identify marker peaks associated with different cell clusters, a Wilcoxon rank-sum test was employed on the PeakMatrix, accounting for biases such as TSS (Transcription Start Site) Enrichment and fragment counts. Marker peaks were considered significant based on criteria set at a FDR of < = 0.05 and a Log2FC of > = 0.58. The FDR was controlled using the Benjamini-Hochberg procedure to reduce the chances of false positives arising from multiple testing. Transcription factor (TF) motif enrichment within the identified marker peaks was assessed using the peakAnnoEnrichment() function. This function performs hypergeometric enrichment of a given peak annotation within the defined marker peaks.

#### Flow cytometry analysis

MFI of the GFP signal for the hspx-high and hspx-low populations at different timepoints was calculated using the software FlowJo (v. 10.9).

#### CFU data

In this study, CFUs were quantified and analyzed to assess differences in bacterial burden between different conditions/treatments. Log10 transformations were applied to each CFU count prior to further analysis. To ensure the appropriateness of parametric tests, the data were first checked for normality using the Shapiro-Wilk test, a method suited for small sample sizes. This test evaluates the hypothesis that a sample comes from a normally distributed population, which is a critical assumption for the application of a two-sample t-test. Further, to assess the equality of variances between the treatment groups, Levene’s test was performed. Upon confirming that the assumptions of normality and homogeneity of variances were satisfied, a two-sample t-test was conducted to determine if there were statistically significant differences in the mean log-transformed CFU counts between the groups. The results of the t-test, including the p-values, were used to infer the statistical significance of the differences observed. The CFU plots presented in the results section are annotated with summary statistics including the mean and standard deviation (SD) of each group, along with the sample size (n), and the p-value from the t-test.

#### Lead Contact and Materials Availability

Further information and requests for resources, reagents and protocols should be directed to and will be fulfilled by the Lead Contact, David G. Russell (dgr8@cornell.edu). This study did not generate new unique reagents. Plasmid, bacterial and mouse strains, antibodies and other reagents and protocols used in this study will be available upon request.

## Data Availability

The datasets supporting the conclusion of this study are available in the Gene Expression Omnibus (GEO) under accession numbers: GSE245950 (scRNA-seq) and GSE245836 (scATAC-seq). The scRNA-seq datasets for the 3-week timepoint and naïve lung were previously published and are available under accession number: GSE167232.
